# Peripheral mononuclear cells composition in platelet-rich fibrin in canines with chronic conditions

**DOI:** 10.1038/s41598-022-22487-4

**Published:** 2022-10-19

**Authors:** Indre Jasineviciute, Juozas Grigas, Gintare Ziukaite, Arnoldas Pautienius, Dainius Razukevicius, Judita Zymantiene, Arunas Stankevicius

**Affiliations:** 1 Department of Anatomy and Physiology, Veterinary Faculty, Lithuanian University of Health Sciences, 47181 Kaunas, Lithuania; 2 Institute of Microbiology and Virology, Lithuanian University of Health Sciences, 47181 Kaunas, Lithuania; 3 Veterinary Faculty, Dr. L. Kriauceliunas Small Animal Clinic, Lithuanian University of Health Sciences, 47181 Kaunas, Lithuania; 4 Department of Maxillofacial Surgery, Faculty of Odontology, Lithuanian University of Health Sciences, 44307 Kaunas, Lithuania

**Keywords:** Inflammation, Lymphocytes, Immunological techniques, Isolation, separation and purification, Cellular imaging, Regeneration

## Abstract

Platelet-rich fibrin (PRF) is a hot research topic because of its regenerative effect in humans. However, data reporting about its application in companion animals is lacking. The study aimed to supplement currently available data on PRF cell composition in canine patients by isolating peripheral blood mononuclear cells (PBMC), namely T cells, matured B cells, monocytes and macrophages, and adapting current protocols of cell flow cytometry for PRF analysis. The canine patient population was divided into three subgroups: animals with periodontitis only, animals with neoplasia and periodontitis, and healthy controls. Individual clinical parameters of the patients and evaluation of the wound healing quality were included in the research. In the present study, canine PRF cell composition was analyzed for the first-time using cell flow cytometry protocol. A higher proportion of PBMC cells related to wound healing (CD3+, CD3+ CD4+ CD8−, CD14+) were found in the PRF of control, periodontitis and neoplasia groups compared to the respective blood samples, which implies a positive outcome associated with clinical PRF usage in canine patients. Proportions of monocytes and macrophages were higher in PRF samples compared to the blood of healthy patients and periodontitis-affected patients. However, inflammatory and neoplastic processes do not affect the distribution of PBMC.

## Introduction

The previous study in dogs and cats revealed that wound healing initiation components of platelet-rich fibrin (PRF), such as levels of different growth factors, are similar to humans^1^. Also, the microscopic appearance of the composition and distribution of PRF seems identical to what was detected in human research^1^. Our research backs up this theory by demonstrating the difference in the levels of peripheral blood mononuclear cell (PBMC) subsets in canines’ PRF. The healing properties of the PRF have previously been evaluated using a variety of study approaches. Recent studies in canines commonly include histological structural analysis or growth factors expression in PRF using enzyme-linked immunosorbent assay^1,2^. However, recent findings are insufficient to support the clinical use of PRF in canines, particularly those with a history of chronic illness.

Our research was based on a new study object—canine patients with chronic inflammation and neoplastic process. Also, it included an adaptation of cell flow cytometry protocol for canine PRF cell composition analysis. Due to the limited flow cytometry analysis in veterinary regenerative medicine, we adapted cell isolation protocol from PRF samples used in human research^3^.

The tissue healing process includes different immune cells: T cells, B cells, macrophages, monocytes, dendritic cells, neutrophils, and other components^4^. T cells are described as a key factor in regenerative therapy^5^. T cell activation is particularly critical during the early stages of wound healing and the scarification process^6^. The significant impact of fast tissue healing is based on T memory cells as the first components that respond to tissue damage and recruit T cells^7^. In the presence of skin injury, T helper cells produce cytokines that regulate inflammatory response activity, re-epithelialization, scarification, and wound contraction. Their involvement in wound healing includes macrophage polarization and increased collagen linking^8^.

Moreover, the role of macrophages in chronic and acute wound healing is the transition of phenotype from pro-inflammatory to anti-inflammatory macrophages^9^. The study model in mice has shown that delayed macrophage transition in the wound site leads to prolonged healing of chronic ulcers^10^. Tissue formation and regeneration are also based on monocyte response and secretion of highly active cytokines, such as TNF-α^8,11^. Another study in mice revealed that the wound healing time might be associated with the direct application of mature B cells that successfully accelerates the tissue regeneration process, including improved parameters like angiogenesis and scar tissue^12^. The main focus of this work was on the features of the cells found in PRF samples, which are directly related to wound healing properties.

It’s worth mentioning that periodontitis and oncological patients are the most common receivers of PRF therapy due to suppressed immune response and slow wound healing process. Additionally, previous studies in PRP showed that the cellular composition of PRP influences growth factor and cytokine concentrations^13^. Moreover, elevated level of pro-inflammatory cells in blood samples of patients is also reflected in the cellular composition of PRP^14^.

Clinical parameters like quality of wound healing parameters and pain score help to evaluate the use of PRF from a practical point of view. Control and recognition of pain in animals is a priority factor directly related to proper analgetic dosage^16^. Increased wound healing quality with less purulent discharge is another crucial characteristic that aids in the avoidance of antibiotic overuse in veterinary medicine.

The study aimed to evaluate the composition of PBMCs in PRF and compare it to whole blood. Another aim was to determine the effect of different chronic illnesses, such as periodontitis and neoplasm, on PBMC subtype proportions and general quality in PRF. Moreover, the regenerative effect of PRF on wound healing in dogs was also evaluated. We hypothesized that PRF is an effective and practical tool which should be considered as a wound treatment option for veterinary patients.

## Results

### Flow cytometry protocol optimization

**Figure 1:**
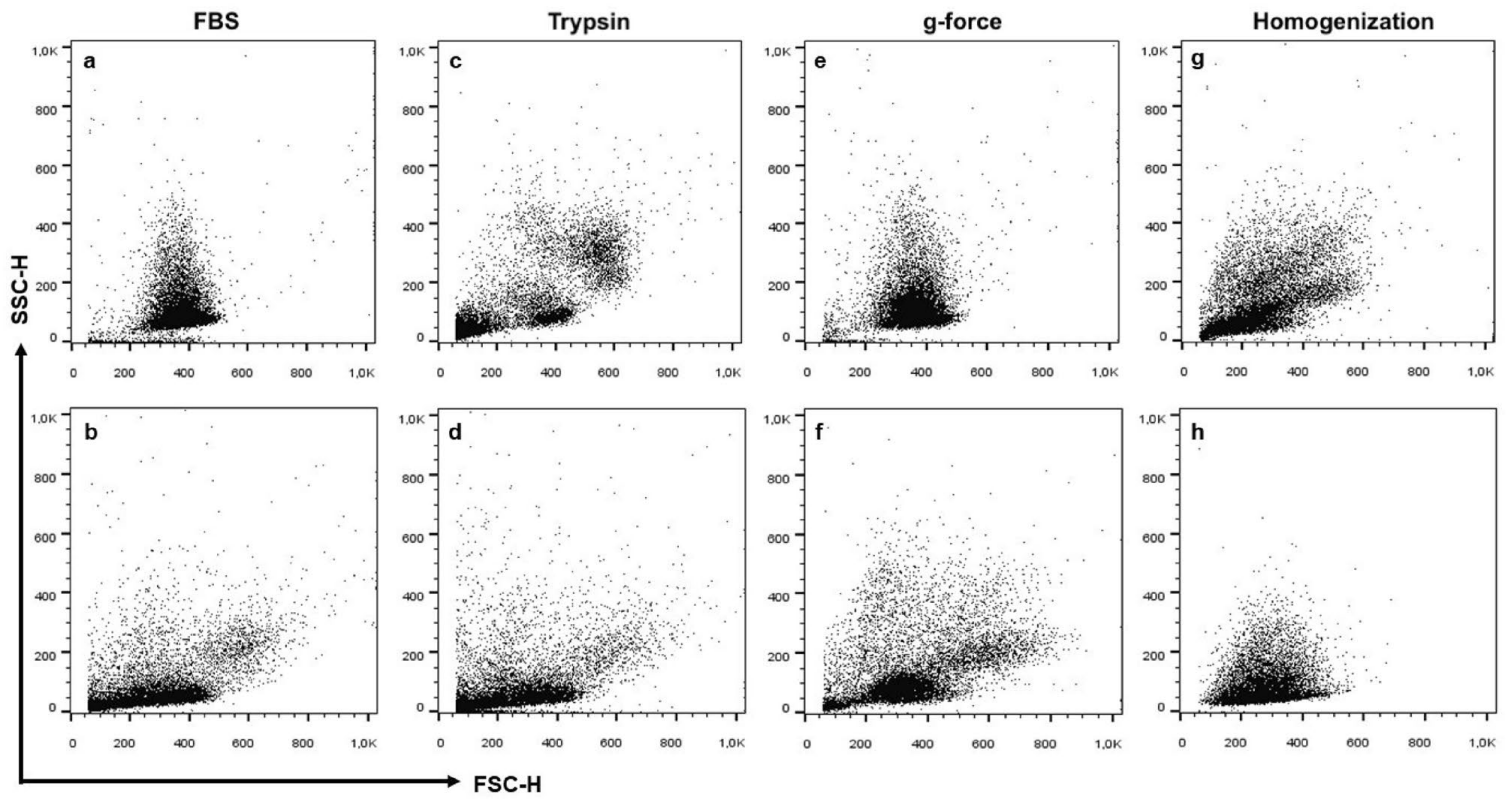
Comparison of cell isolation from PRF samples using different protocols for flow cytometry analysis. (**a**) Incubation with FBS; (**b**) without FBS; (**c**) incubation with trypsin; (**d**) without incubation with trypsin; (**e**) centrifugation g-force 700 g; (**f**) reduced centrifugation g-force 500 g; (**g**) homogenization after adding PBS; (**h**) homogenization before adding PBS.

A modified protocol of PBMC extraction from whole blood samples was adapted to PBMC extraction from PRF samples. Regarding the quality of flow cytometry analysis results, the technique with the fewest artefacts, the most apparent separation of PBMC subsets, and the fewest red blood cells was chosen (Fig. 1).

Homogenization and mincing of the PRF sample before adding phosphate buffer (PBS) helped to separate cells within the fibrin mesh and remove it from further steps of cell isolation (Fig. 1g,h). Incubation with trypsin (Fig. 1c,d) and fetal bovine serum (FBS) (Fig. 1a,b) did not improve the quality of PMBC isolation and were not useful in the removal of fibrin particles. Centrifugation g-force was reduced from 700 to 500 g for a lesser impact on cell vitality, resulting in more viable cells for subsequent flow cytometry analysis (Fig. 1e,f). The mean of viable cells using 700 g parameter was 83.82% (SE = 3.77, CI 95% 63.8–97.6) compared to increased viability of 90.5% (SE = 1.12, CI 95% 87.9–93.1) using 500 g parameter.

### T cells subset proportions are higher in PRF samples compared to whole blood of healthy controls and periodontitis patients

Cell flow cytometry has been used to assess PBMC subset distribution differences in whole canine blood and PRF samples. T cells subsets (CD3+, CD3+ CD4+ CD8+, CD3+ CD4+ CD8−, CD3+ CD4− CD8+, CD3− CD8+), monocytes and macrophages (CD14+), and mature B cell (CD21+) proportions were calculated as percentages of viable cells.

**Figure 2:**
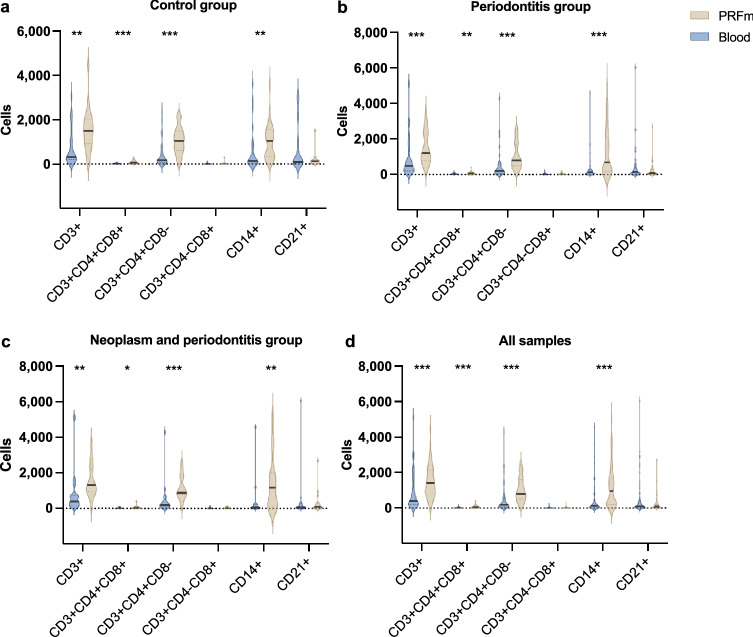
Comparison of PBMC groups between whole blood (blue) and PRF samples (brown) in three patient groups: (**a**) control, (**b**) periodontitis, and (**c**) neoplasm and periodontitis. PBMC distribution of all samples is presented in (**d**). *p < 0.05, **p < 0.01, ***p < 0.001.

CD3+ cells were referred to as T cells of the viable cell population. A significantly higher proportion of CD3+ cells was observed in PRF (17.6%) of healthy canine patients compared to whole blood samples (9.97%, *p* < 0.01) (Fig. 2a). A similar tendency of higher CD3+ cells proportion in PRF samples (17.64%) compared to whole blood samples (11.25%, *p* < 0.01) was revealed in periodontitis affected patients (Fig. 2b).

Figure 2a,b demonstrate that CD3+ CD4+ CD8− (T helper) cells had a significant proportion change in PRF compared to whole blood of both the control group (from 56.6% to 70.35%, *p* < 0.05) and periodontitis group (from 49.03% to 68.16%, *p* < 0.001).

In contrast, there was no difference in the proportion of CD3+ CD4+ CD8+ (T memory cells), CD3+ CD4− CD8+ (T cytotoxic cells) and CD3− CD8+ (NK cells) T cells subtypes in the PRF of healthy dogs and patients with periodontal disease. PMBC distribution of healthy and periodontitis canine groups can be found in Tables S1 and S2.

### Monocyte and macrophage proportions are higher in PRF samples compared to whole blood of healthy controls and periodontitis patients

Our study revealed that the proportion of CD14+ was higher in PRF samples (8.01%) in healthy canines compared to their respective whole blood samples (12.05%, *p* < 0.01) (Fig. 2a). The difference in CD14+ distribution was much more substantial in periodontitis patients’ samples compared to healthy controls (*p* < 0.001) (Fig. 2b).

### CD21+ cell proportions in PRF and whole blood of healthy controls and periodontitis patients

The proportion change of CD21+ (mature B cells) was not more remarkable in PRF samples compared to the whole blood of the control and periodontal disease groups. CD21+proportion in PRF samples (2.21%) does not make a significant change compared to whole blood (8.98%, *p* > 0.05) in the healthy controls group (Fig. 2a). The same result was found in the periodontitis group where mature B cells distribution was 6.11% in whole blood and 3.27% in PRF samples (*p* > 0.05) (Fig. 2b).

### Inflammatory and neoplastic processes do not affect the proportion distribution of PBMC subtypes in PRF

**Figure 3:**
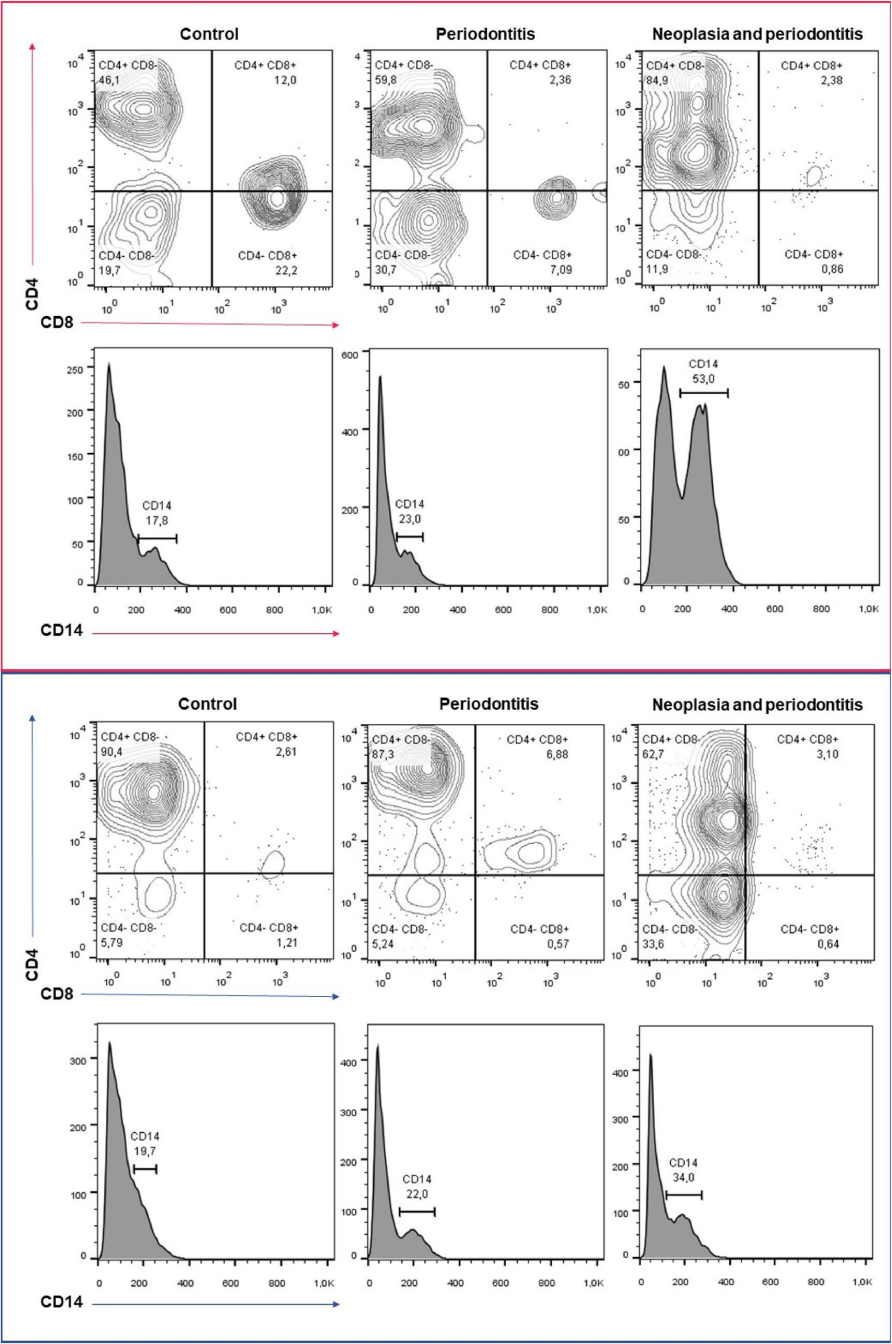
PBMC substitutes CD4+ CD8−, CD4+ CD8+, CD4− CD8−, CD4− CD8+ in whole blood samples (marked red) and PRF samples (marked blue) from the top left: 1—control, 2—periodontitis, 3—neoplasm and periodontitis. Cells were gated for expression of CD4+ CD8+, CD4+ CD8−, CD4− CD8−, and CD4− CD8+. CD14+ PMBC subsets are visualized in the histogram. Cells were gated for expression of CD14+, which is expressed in percentage.

Periodontitis patients were separated into subgroups with and without an underlying neoplastic condition to assess the impact of the neoplastic process on the proportion distributions of PBMC subtypes in PRF. No significant changes in PBMC subtype proportions were observed between whole blood and PRF samples of periodontitis patient subgroups (Fig. 3). Patterns of PBMC subtype distribution were similar between periodontitis subgroups and the control group (Fig. 2b).

Additional analysis was performed to assess the composition of PRF in the periodontitis and neoplastic process patient groups, which demonstrates that the subtypes of cells that are increased in PRF are similar to those found in the control and periodontitis groups (Fig. 2c). Significant composition changes were found in CD3+, CD3+ CD4+ CD8− and CD14+ PBMC subsets. The significance is even more decisive in comparison of all samples (Fig. 2d). PMBC subtype distribution in neoplasia and periodontitis subgroups can be found in Tables S3 and S4.

### Factors such as age, weight, sex, skull type and stage of periodontitis do not affect the proportion distribution of PBMC subtypes

**Figure 4:**
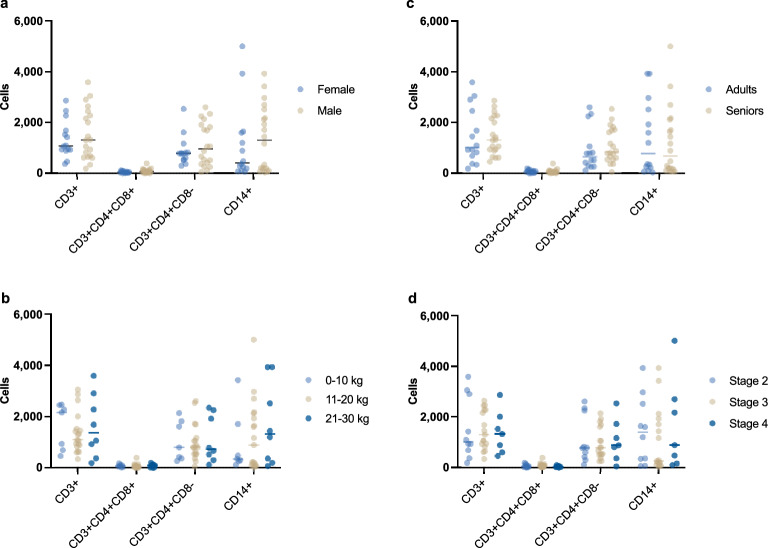
Relation between PMBC subsets and different characteristics of patient groups: (**a**) sex, (**b**) weight, (**c**) age and (**d**) periodontitis stage.

Individual and clinical features analysis that included patients’ age and weight group, sex, skull type and periodontitis stage did not show a significant impact on PBMC subtype proportion distributions (Fig. 4). A weak relationship between weight and distribution of CD3+ (*p* = 0.05), CD3+ CD4+ CD8+ (*p* = 0.027), and CD14+ (*p* = 0.006) subtypes was observed in whole blood samples, but not in PRF. Another weak relationship in whole blood samples was found between skull types and CD14+ (*p* = 0.034), CD3+ CD4+ CD8+ (*p* = 0.034), as well as periodontitis stages three and four and CD3+ CD4+ CD8+ (*p* = 0.044).

### Early wound healing scale and pain scale results

**Figure 5:**
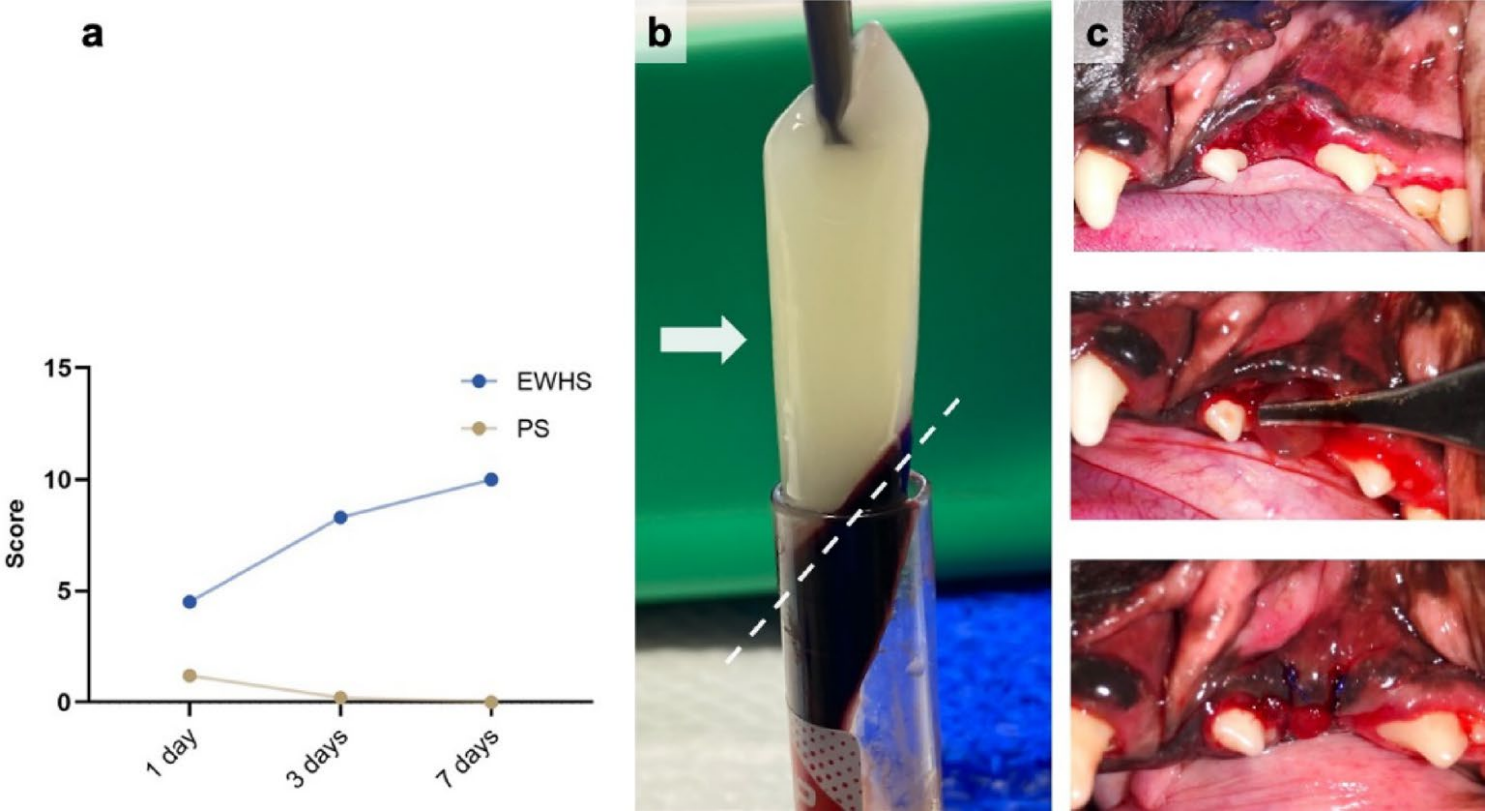
(**a**) Results of average EWHS and PS after one, three and seven days in patients with PRF included in the post-extraction wound site. (**b**) PRF sample (marked with arrow) and separation site from the bottom layer of red blood cells (marked with dashed line). (**c**) PRF membrane positioning and fixation in post-extraction wound site after creating a mucosal flap.

The average EWHS in patient group were 4.5 and 8.3, 24 and 72 h post-PRF application, respectively. In every case, the maximum score of 10 was observed after seven days post-PRF application, demonstrating total wound healing. Pain scale results were acquired from the survey. The average score of PS after 24 h was 1.2; after three days, it was 0.2, and no signs of pain were detected after one week. A summary of these results is presented in Fig. 5.

## Discussion

The present study aimed to evaluate the cellular composition of canine PRF and compare it to the whole blood, in addition to observing how different chronic diseases, such as periodontitis and neoplastic process, affect the proportions of PBMC subtypes in PRF. Furthermore, we attempted to examine PRF therapy’s impact on healing a post-extraction wound in the oral cavity.

Due to limited data about PBMC extraction from PRF samples for flow cytometry analysis, the blood samples preparation protocol was used as a template. Flow cytometry is an essential tool for PBMC evaluation, even though the enzyme-linked immunosorbent assay has been employed more frequently when evaluating the cellular composition of PRF^1,2^. Mincing with scissors or a scalpel blade before diluting the PRF sample with PBS resulted in more effective fibrin removal and a higher yield of viable cells. We opted not to use tissue homogenizer due to a negative effect on cell viability, as previously suggested^3^. In authors’ opinion, using additional components, such as fetal bovine serum and trypsin, did not impact the yield of the extracted PBMCs^3^. Moreover, enzymes like trypsin may negatively impact immunophenotyping due to its cell membrane protein-altering qualities^3^.

Significantly higher proportions of CD3+, CD3+ CD4+ CD8− and CD14+ PBMC subtypes were observed in PRF samples compared to whole blood samples of every patient group, representing populations of T memory cells, T helper cells, macrophages, monocytes and dendritic cells, respectively. An increase in the proportions of cells mentioned above in PRF may be associated with the regenerative qualities of PRF.

Macrophages and monocytes contribute to angiogenesis and play an essential role in immune response and tissue repair^17,18^. Toll-like receptor signalling is expressed by dendritic cells and macrophages, which aids in the production of inflammatory cytokines such as IL-1, IL-6, and TNF-α. Lymphocytes are known for specific and nonspecific roles in the tissue healing process, even though they are not prominent players in the first stage of the tissue repair process^4^. An increase in macrophage/monocyte and T cell proportions in PRF may contribute to faster wound healing in cases of PRF application.

PBMC subtype changes observed in PRF compared to the whole blood may be due to the ability of fibrin and fibrinogen to interact with white blood cells (WBC) by binding cell surface receptors within the matrix of PRF. Fibrin tends to form a complex with fibrinogen, which eventually binds to WBC^19^. An important role here is played by ligands of the β integrin family, which are expressed by various cells, including lymphocytes, macrophages, and monocytes. Furthermore, previous studies demonstrated similar findings in different animal species and humans^20^. Fibrinogen and fibrin engagement with WBC are significant for inflammatory response and clotting initiation^21,22^. The relationship between fibrin and cells is vital in creating a three-dimensional fibrin matrix in PRF that keeps cells and bioactive molecules trapped.

Additionally, PRF consists of cytokines, glycan chains, structural glycoproteins, and growth factors that are enmeshed within the fibrin network and impact extracellular matrix proteins, which are responsible for migration, division, and endothelial cell phenotype change^22^. Components involved in PRF structure, such as different cell types, bioactive molecules, and fibrin matrix with fibronectin and vitronectin, are the key to accelerating and assisting the wound healing process. A higher concentration of lymphocytes in the PRF membrane demonstrates its potential to keep WBC within the fibrin mesh.

No differences were observed in PBMC subtype distribution between the control and patient groups. These results demonstrate that neoplastic or chronic inflammatory processes do not affect the proportions of PBMCs in PRF. Previous studies in humans concluded that a general decline in the number of distinct lymphocyte subsets in the blood of neoplasm patients was observed^23,24^. In another study, cancer stage and type were described as one of the critical factors affecting CD4+, CD8+ and NK cell proportions^25^. Angiogenetic features of PRF concern the development of vascularisation of the tumor, which is one of the main steps of the metastatic cascade. On the other hand, the long-term analysis with a high number of breast cancer patients showed no local oncogenic potential of PRP, which seems safe in high-risk cancer patients^26^.

Comparison of PBMC subtypes in PRF of periodontitis patients with and without underlying neoplastic process demonstrates that CD3+, CD3+ CD4+ CD8+, CD3+ CD4+ CD8− and CD14+ proportions did not differ between PRF samples of patient’s subgroups. A previous study described the association between periodontitis and the bioactive cancer process, which can be the key to similarities in cell distribution in whole blood and PRF^27^. The chronic inflammatory process of periodontal disease with constant immunological stimulation and oxidative stress leads to the systematic effect and neoplastic process development^27^. In human studies, a significantly higher proportion of intermediate monocytes and macrophages was detected in inflamed gingival tissue compared to healthy tissues and periodontal disease affected tissues^28^. Even though chronic inflammation plays a role in T cell differentiation, reactive oxygen species can diminish the response, which is especially important in tumour growth. T cell response is also highly dependent on the location of lymph node involvement^29^, which leads to the idea that even though bioactive processes impact T cells, the general distribution of PBMC might stay the same in different subgroups because of different affecting factors like inflammation or neoplasia site.

Different individual clinical parameters were evaluated to find their effect on PRF composition. The impact of age on PMBC distribution was evaluated due to the studies where aging was determined as one of the factors that affect lymphocyte differentiation balance (naive and memory T cells shift) in humans^30^. Even though studies in mice and humans revealed that levels of oxidized compounds produced by lymphocytes increase with age in our study, age and sex were not statistically significant factors affecting PBMC subtype proportions in PRF^29^. Lymphocyte and cytokine levels can be affected by factors like the patient’s environment, genetics or hormones. In one study, it was revealed that overall cytokine level was higher in men compared to women, which is related to molecular weight and size that allows it to be entrapped in fibrin matrix. Unfortunately, no research has been done on the effect of sex, age and weight on lymphocytes in animals’ PRF. Weight gain in humans has also been associated with changes in lymphocyte distribution. Levels of T memory cells increased with a higher body mass index^32^. However, our study was limited in its ability to thoroughly analyse the influence of weight disparities on lymphocyte subgroup percentage due to a lack of body mass index analyses and samples from obese patients.

Periodontitis is a chronic inflammation of periodontal tissue which systematically affects an organism, stimulating bone marrow to release more WBC into the blood. There is a link between severeness of periodontitis and WBC count^33–35^. The involvement of T cells in periodontitis progression has been studied in both human and animal models. The optimal balance of T cell subsets like CD4+, CD8+, T helper cells and NK cells is crucial for healthy periodontal tissue^34^. Fibrin network density studies demonstrate that fibrin is looser and denser in patients with periodontitis and in older patients^36^. Contrary to the previous finding, this study did not find a relationship between changes in PBMC subtype proportions and periodontal disease stage. An analysis of human patients demonstrated that periodontal condition does not affect the healing potential of platelet-rich fibrin^37^.

PRF has previously been used as an effective pain management tool in humans’ early stages following tooth extraction. However, the long-term effect is still not clear^38,39^. Early wound healing with PRF showed beneficial results in reduced swelling, lower pain level and higher healing index^40,41^. Our data is consistent with previous findings, suggesting the benefits of PRF use in post-extraction pain management and wound healing^41^.

While our findings support the practical side of platelet-rich fibrin membrane use in canine patients with a different clinical background, these results should be interpreted with caution due to study limitations such as the relatively small sample size, especially in the neoplastic patient group and post-surgical evaluation group. Grading and staging of malignant neoplasms was not performed in this study, preventing us from drawing conclusions about potential differences in cell subset distributions between patients with non-invasive and metastatic neoplasms. Moreover, more parameters of lymphocyte detection by flow cytometry should be included to confirm cells as the specific type of T cell subsets.

## Materials and methods

### Animal rights statement

All tests of canines met welfare and ethical standards for animal research by the ARRIVE guidelines. The procedures with canine patients were performed after obtaining informed consent and signed approval from official owners or guardians. The Ethics Commission of Lithuania approved procedures under the State Food and Veterinary Service (Decision no. G2-127/2019) and we confirm that all experiments were performed in accordance with relevant guidelines and regulations.

### Patients’ characteristics

Our study included 59 samples of whole dog blood and the same number of PRF samples, of which 25 were clinically healthy canines as the control group and 34 canine patients diagnosed with periodontitis (*n* = 17) or periodontitis with an underlying neoplastic process (*n* = 17). Epithelial origin, lymphoid tissue and connective tissue tumours were included in our study (Table S5). Squamous cell carcinoma, usually diagnosed in mammary glands, was the most common tumour among patients (*n* = 6). Histopathological test results confirmed neoplasm diagnosis. Most of the canines in both groups had a mesocephalic skull structure. The stage of periodontitis was evaluated by clinical parameters based on AAHA Dental care guidelines: alveolar pockets depth was measured with a dental probe in six sites around the tooth, gingival inflammation, plaque and calculus accumulation, and dental radiograph results. Periodontitis stage 1 was absent in in-patient groups. Detailed patient characteristics are described in Table 1.

**Table 1:** Characteristics of the control group and canine periodontitis and neoplasia group.

Variables	Patients (*n* = 34)	Control group (*n* = 25)
**Age, y/o**		
0–8 (adult)	14	22
9–17 (senior)	20	3
**Sex**		
Female, *n* (%)	14 (41.2)	14 (56)
Male, *n* (%)	20 (58.8)	11 (44)
**Skull type**		
Mesocephalic, *n* (%)	27 (79.4)	24 (96)
Brachycephalic,* n* (%)	4 (11.8)	1 (4)
Dolichocephalic, *n* (%)	3 (8.8)	0 (0)
**Weight, kg**		
0–10, *n* (%)	7 (20.6)	13 (52)
11–20, *n* (%)	19 (55.9)	6 (24)
21–30, *n* (%)	8 (23.5)	6 (24)
Neoplasia patient, n (%)	17 (50)	
**Periodontitis stage**		
2, *n* (%)	10 (29.4)	
3, *n* (%)	17 (50)	
4, *n* (%)	7 (20.6)	

### Collection of blood samples and preparation of PRF

A whole blood sample was collected from a cephalic vein into a sterile 8.5 ml glass tube with acid citrate dextrose (BD Vacutainer ACD A Blood Collection Tube, Becton, Dickinson and Company, Franklin Lakes, NJ, USA) and was stored at 4 °C temperature for further analysis. For the preparation of the PRF, another 10 ml of autologous blood sample was collected from a cephalic vein into a 10 ml sterile glass tube without anticoagulant (Dr Choukroun Glass A-PRF Tubes, Auckland, New Zealand) and immediately centrifuged. PRF was obtained by a fixed-angled centrifuge using 700 g-force for 12 min at room temperature. PRF sample was gently separated 2 mm below the connection to the bottom layer of red blood cells and was stored at 4 °C temperature until further evaluation.

### Protocol optimization trial

Additional steps were applied to adapt the PRF samples preparation protocol for flow cytometry analysis. FBS (200 µl, 37 °C, 15 min) was added after the homogenization of PRF. Another trial included incubation with trypsin (1 ml, 37 °C, 5 min) after homogenization of PRF. Furthermore, changes of the g-force were applied and reduced from 700 to 500 g in the sample centrifugation step, as well as different homogenization procedures adding PBS before and after homogenization of the PRF.

### Surgical procedure and evaluation of healing and pain parameters

An additional part of the study included a group of 10 canine patients that had periodontitis or periodontitis combined with neoplasm. The procedure was done under general anesthesia. Before surgery, the oral cavity was cleaned with chlorhexidine 0.12% solution. After, PRF was prepared using the protocol above. After separation and compression, the PRF membrane was placed and fixated in the post-extraction dental wound. A mucosal flap was created and sutured to close the wound. A control visit was made on the third and seventh days after the procedure. Post-surgical analgesia was 0.1 mg/kg of oral meloxicam once a day for three days. The early wound healing^42^ and Colorado State University pain score protocol was adapted for the healing effect evaluation, and information was collected from the owners following a survey. Early wound healing scale included clinical signs of epithelization (merging of incision margins), inflammation (redness and swelling) and hemostasis (bleeding and presence of fibrin). Scoring was carried out by evaluating Epithelization (0–6 points) and other parameters (0–2 points). Pain scoring was carried out using a survey which included parameters of patient behavior, such as body expression (position of eyes, ears), vocalization, responsiveness, activity, and appetite. The result was evaluated on a 0–3 point scale.

### Flow cytometry analysis

Whole blood samples were diluted with 5 ml of PBS. Peripheral blood mononuclear cells (PBMC) were isolated using Ficoll-Paque PLUS (GE Healthcare, Chicago, IL, USA) according to the manufacturer’s instructions. After density gradient centrifugation at 4 °C temperature. PBMCs were suspended in 1 × PBS. PBMCs were washed three times and resuspended to 1 × 10^6^ cells/ml concentration. PRF samples were minced with a scalpel blade and diluted with 8 ml of phosphate buffer. After centrifugation (500 g), supernatants (PRF extracts) were collected, and samples were washed three times with PBS and resuspended to 1 × 10^6^ cells/ml concentration. PBMCs from whole blood and PRF samples were used for staining immediately after isolation for 30 min. Fluorescence markers and antibodies used for PBMC staining are presented in Table 2. Following the staining procedure, all samples were washed twice with PBS and resuspended in 1 × PBS. Cytometry data was collected by using the FACSCalibur flow cytometer (BD Biosciences, Franklin Lakes, NJ, USA) and acquiring 10,000 events for each sample, followed by data analysis using FlowJo v10 (Tree Star) software.

**Table 2:** Antibodies were used in the characterization of different PMBC subsets in canine.

Target	Target species	Conjugate	Antibody clones	Reactivity
CD3e	Canine	FITC	CA17.2A12	T cells
CD4	Canine	FITC	YKIX302.9	T-helper cells
CD8a	Canine	PE	YCATE55.9	T-cytotoxic cells
CD14	Universal	FITC	TüK4	Monocytes and macrophages (some types)
CD21	Canine	PE	LT21	Mature B cells

**Figure 6:**
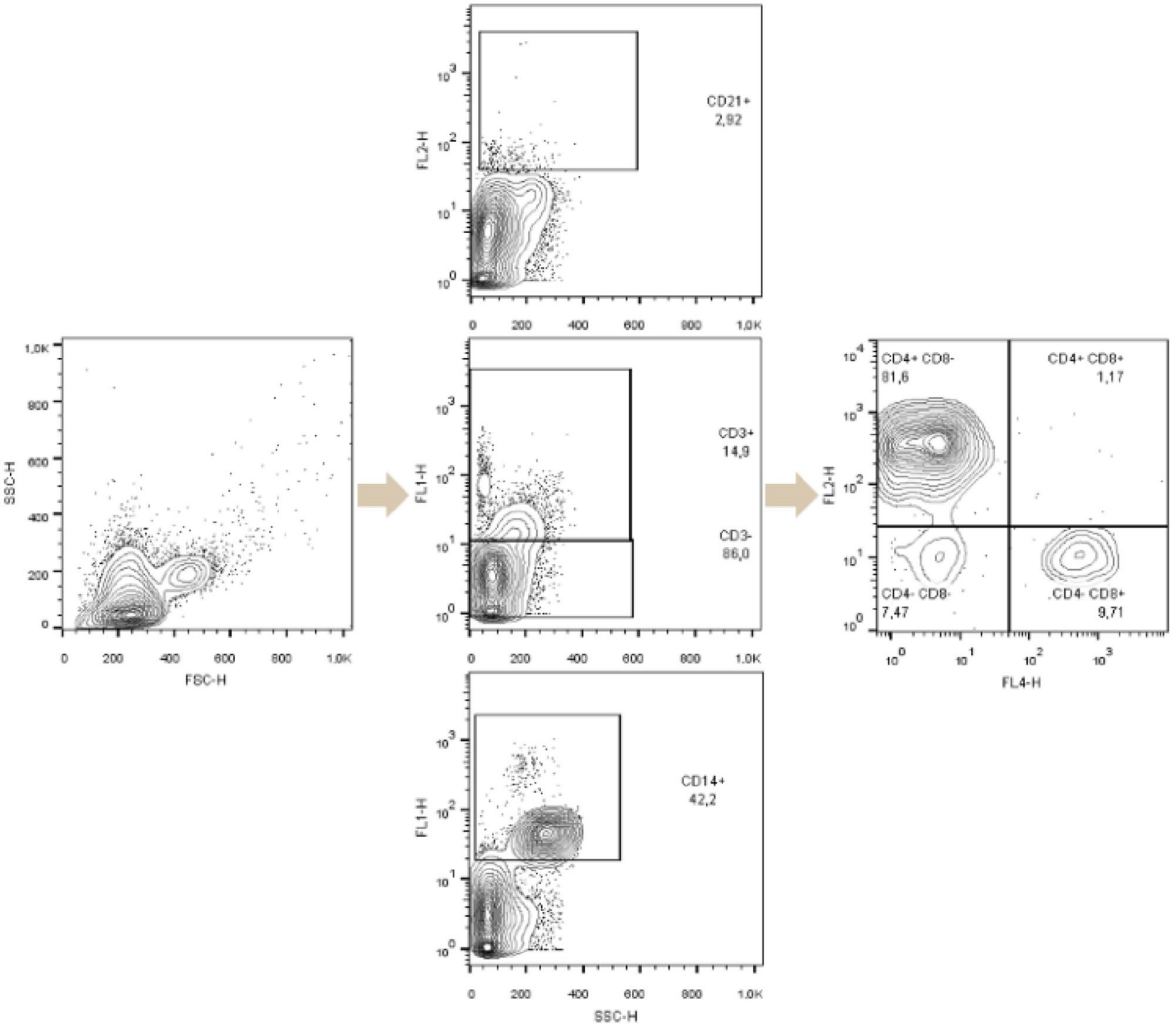
Gating strategy applied to flow cytometry data. Viable cells were selected by FSC and SSC gate. Other gates based on CD3+, CD3−, CD14+ and CD21+ markers were established within viable cell gates. CD4+ CD8−, CD4+ CD8+, CD4− CD8−, CD4− CD8+ were established within CD3+, CD3− gate.

CD3+ and CD3−, CD14+ and CD21+ cell gates were established within the viable cell population. CD4− CD8+ (T cytotoxic cells), CD4+ CD8+ (memory T cells), CD4+ CD8− (T helper cells) gates were established within the CD3+ cell population. All T cell population sizes were expressed as proportions of CD3+ cells and total viable cells. CD14+ (macrophages and monocytes) and CD21+ (mature B cells) gates were established within the viable cell population and expressed as a proportion of the total cell population. The gating strategy used in this study is summarized in Fig. 6.

### Statistical analysis

GraphPad Prism 9 was used for statistical analysis. To assess differences between patients and the control group as well as between different characteristic subgroups Mann–Whitney-U test was performed. Values *p < 0.05, **p < 0.01, and ***p < 0.001 considered statistically significant.

## Supplementary Information

Supplementary Information.

## Data Availability

The datasets generated during and analysed during the current study are available from the corresponding author upon reasonable request.
