# Supplementary material for: Peripheral mononuclear cells composition in platelet-rich fibrin in canines with chronic conditions
Source: Sci Rep. 2022 Oct 19;12:17426. doi: 10.1038/s41598-022-22487-4 (PMC9582024; doi:10.1038/s41598-022-22487-4)
Supplement: Supplementary file 1 — Supplementary Information. [file 41598_2022_22487_MOESM1_ESM.pdf]

Peripheral mononuclear cells composition in platelet-rich fibrin in canines with chronic conditions

Indre Jasineviciute*, Juozas Grigas, Gintare Ziukaite, Arnoldas Pautienius, Dainius Razukevicius, Judita Zymantiene, Arunas Stankevicius

Table S1. PMBC subsets in control group and canines affected by periodontitis whole blood and PRF samples.

Cell subsets	Blood			PRF		
	Control, (*n*=25) Mean Statistic	Periodontitis, (*n*=34) Mean Statistic	*p* value	Control, (n=25) Mean Statistic	Periodontitis, (n=34) Mean Statistic	*p* value
CD3+	754.88	882.65	0.662	1478.04	1466.56	0.881
CD3+, CD4+, CD8+	24.12	36.94	0.425	82.38	69.82	0.399
CD3+CD4+CD8-	473.48	530.74	0.872	1027.5	1028.38	0.748
CD3+CD4-CD8+	20.60	37.50	0.174	37.65	37.79	0.433
CD14+	618.68	393.97	0.167	999.81	1278.97	0.952
CD21+	699.40	483.15	0.326	165.69	260.41	0.771

Table S2. Comparison of PMBC subsets in PRF and whole blood samples in control group and canine patients affected by periodontitis.

Lymphocyte subsets	**Control (*n*=25), Means**			**Periodontitis (*n*=34), Means**		
	Blood	PRF	*p* value	Blood	PRF	*p* value
CD3+	754.88	1478.04	**0.003**	882.65	1466.56	**<0.001**
CD3+CD4+CD8+	24.12	82.38	**<0.001**	36.94	69.82	**0.028**
CD3+CD4+CD8-	473.48	1027.5	**<0.001**	530.74	1028.38	**<0.001**
CD3+CD4-CD8+	20.60	37.65	0.365	37.50	37.79	0.663
CD3-CD8+	8.92	16.77	0.909	25.41	63.56	0.270
CD14+	618.68	999.81	**0.004**	393.97	1278.97	**<0.001**
CD21+	699.40	165.69	0.194	483.15	260.41	0.492

Bold values indicate statistically significant values ($p<0.05$)

Table S3. PMBC subsets in neoplasia group and canines affected by only periodontitis whole blood and PRF samples.

Cell subsets	**Blood**			**PRF**		
	Neoplasia and periodontitis, (*n*=17), Mean Statistic	Periodontitis, (*n*=17) Mean Statistic	*p* value	Neoplasia and periodontitis, (*n*=17), Mean Statistic	Periodontitis, (*n*=17) Mean Statistic	*p* value
CD3+	798.24	967.06	0.593	1514.59	1418.53	0.563
CD3+, CD4+, CD8+	24.24	49.65	0.098	69.94	69.71	0.757
CD3+CD4+CD8-	524.35	537.12	0.959	1136.12	920.65	0.102
CD3+CD4-CD8+	30.35	44.65	0.301	37.65	37.94	0.918
CD14+	423.29	364.65	0.535	1395.71	1162.24	0.959
CD21+	487.18	479.12	0.469	341.94	178.88	0.946

Table S4. PMBC subsets concentration in periodontitis and neoplasm patient group samples of whole blood and PRF.

Lymphocyte subsets	**Neoplasm and periodontitis (*n*=17), Means**		
	Blood	PRF	*p* value
CD3+	798.24	1514.59	**0.002**
CD3+CD4+CD8+	24.24	69.94	**0.018**
CD3+CD4+CD8-	524.35	1136.12	**<0.001**
CD3+CD4-CD8+	30.35	37.65	0.501
CD3-CD8+	7.35	47.24	0.170
CD14+	423.29	1395.71	**0.004**
CD21+	487.18	341.94	0.823

Table S5. Distribution of different neoplasia types among patient group.

Tissue of origin and tumor	Number of patients (*n*=17)
**Epithelial origin**	
Malignant	
Adenocarcinoma	3
Squamous cell carcinoma	6
Benign	
Adenoma	2
**Lymphoid tissue**	
Lymphoma	1
**Connective tissue and derivatives**	
Malignant	
Soft tissue sarcoma	3
Benign	
Lipoma	2
